# Recalibrating cell fate: targeting the mitochondrial signaling hub with natural active compounds to inhibit regulated cell death in diabetic kidney disease

**DOI:** 10.3389/fphys.2026.1774714

**Published:** 2026-03-23

**Authors:** Yinzhong Dai, Chenguang Wu, Jiaying Zheng, Keqin Zhao, Shimei Hua, Jianing Sun, Han Zhu, Jun Luo, Junwei Shi, Lu Han, Lifan Wang, Peng Liu

**Affiliations:** 1 Renal Division, Department of Medicine, Heilongjiang Academy of Chinese Medicine Sciences, Harbin, China; 2 Renal Division, Xiyuan Hospital, China Academy of Chinese Medical Sciences, Beijing, China; 3 Beijing Engineering Research Center of Printed Electronics, Beijing Institute of Graphic Communication, Beijing, China

**Keywords:** apoptosis, diabetic kidney disease, ferroptosis, mitochondria, phytochemicals, pyroptosis, regulated cell death

## Abstract

Diabetic kidney disease (DKD) is the leading cause of end-stage renal disease worldwide. The progression of DKD is closely related to various cell death (RCD) pathways such as apoptosis, pyroptosis and ferroptosis. Although historically viewed as distinct events, we propose that mitochondria function as the central hub integrating hyperglycemic, lipotoxic, and pro-inflammatory insults. We delineate how initial hyperglycemic and hemodynamic insults compromise mitochondrial quality control, triggering a vicious cycle: dysfunctional mitochondria release ROS and damage-associated molecular patterns to initiate regulated cell death and inflammation, which in turn further impairs mitochondrial bioenergetics, thereby amplifying diabetic kidney injury. Mechanistically, mitochondrial outer membrane permeabilization triggers intrinsic apoptosis, while the cytosolic leakage of mitochondrial reactive oxygen species (mtROS) and mitochondrial DNA (mtDNA) primes the NOD-like receptor family pyrin domain containing 3 (NLRP3) inflammasome to drive pyroptosis. In parallel, organelle-level metabolic and redox instabilities fuel the lipid peroxidation characteristic of ferroptosis. We highlight the sophisticated crosstalk within this network, such as the Caspase-3/Gasdermin E switch, arguing that these pathways function as a network of molecular crosstalk and functional interdependence with distinct spatiotemporal dynamics, rather than a singular execution program. Regarding therapeutic interventions, we summarize preclinical evidence for natural active compounds like berberine and quercetin. These phytochemicals act as network-level modulators of mitochondrial targets to restore cellular homeostasis. Finally, we critically address the “translational gap” posed by poor oral bioavailability and lack of human target validation. We also explore emerging biophysical concepts, such as liquid-liquid phase separation, as a speculative yet novel frontier for organizing pathological metabolic signals. Therefore, disrupting this mitochondrial feedback loop, when coupled with advanced delivery strategies, represents a strategic therapeutic avenue to arrest DKD progression.

## 1 Introduction

Diabetic kidney disease (DKD) currently stands as the leading cause of end-stage renal disease globally, posing a significant burden on public health systems (Zhao et al., 2018; Liu et al., 2024; He et al., 2025). However, even with the clinical practice of intensive glycemic control, blockade of the renin-angiotensin system and sodium-glucose cotransporter 2 inhibitors (SGLT2), a significant percentage of patients will continue to experience renal deterioration (Rout, ). It is this residual risk that implies an exclusive emphasis on systemic metabolic and hemodynamic alterations may not be able to address the intracellular signaling derangements driving the erosion of renal architecture (Tang et al., 2025). Consequently, exploring direct pharmacological modulation of the mitochondrial network may offer a complementary approach to current therapies. The review offers a framework of assessing phytochemicals as regulated cell death (RCD) pathway modulators, instead of non-specific antioxidants by defining mitochondria as a signaling nexus of various RCD pathways.

Historically, renal cell loss in DKD was attributed primarily to apoptosis. However, the pathological landscape is far more heterogeneous, involving inflammatory and oxidative death modalities such as pyroptosis and ferroptosis (Fang et al., 2025). These RCD pathways are not fixed; they change with the progression of the disease (Fan et al., 2024; Jin et al., 2024). Although the pre-eminent role of apoptosis is evident in the initial phases of tubular hypertrophy and podocyte loss, very inflammatory mechanisms such as pyroptosis and ferroptosis play a central role in the shift to tubulointerstitial fibrosis in the late stages. Instead of considering them as independent parallel phenomena, this review assumes that they represent integrated products of a concerted cellular response to stress (Mao et al., 2025).

The kidney is a high-energy-demand organ that depends upon efficient oxidative phosphorylation in mitochondria to facilitate active transport and reabsorption via the tubules. This metabolic balance is derailed in the diabetic milieu: the chronic hyperglycemia and the lack of the ability to burn the fatty acids causes a maladaptative change in the renal metabolism. The heart of this integration is the mitochondria which act as a metabolic and signaling nexus in the diabetic kidney (Liu et al., 2022). Instead of being the linear upstream switch, however, mitochondria serve as central integrators within complex feed-forward loops (Li et al., 2025). Initially impaired through hyperglycemia-induced oxidative stress and lipotoxicity, impaired mitochondria liberate reactive oxygen species (mtROS) and damage-associated molecular patterns (DAMPs) (Deng et al., 2025). These messages cause controlled cell death and inflammation, which in turn also further impede mitochondrial bioenergetics, forming a positive feedback loop of kidney damage. Thus, the diverse RCD modalities are distinct phenotypic manifestations of this fundamental, mitochondria-centric checkpoint failure.

Plant-based bioactive compounds, or phytochemicals, offer a possible multi-target approach with the capacity to shape up these complex pathological networks (Geng et al., 2023; Zhong et al., 2024; Tian et al., 2024; Zhao et al., 2024). However, these agents cannot be simply grouped as either antioxidants or anti-inflammatory agents, as the actual biophysical processes behind their effectiveness are not as simple (Niazpour and Meshkani, 2025). To address structural heterogeneity, we propose that phytochemical efficacy is governed by distinct biophysical principles: alkaloids like berberine function as lipophilic cations permeating the mitochondrial matrix, while polyphenols like quercetin utilize redox-active moieties to act as electrophilic sensors. Thus, despite diverse structures, these compounds functionally unify as ‘mitochondrial network modulators,’ intervening through either direct targeting or upstream redox signaling. As detailed in the subsequent sections, we reframe these compounds as network-level modulators that interact with key nodes in the mitochondrial death machinery to restore cellular homeostasis.

## 2 Mitochondrial mechanisms of RCD

**Figure 1:**
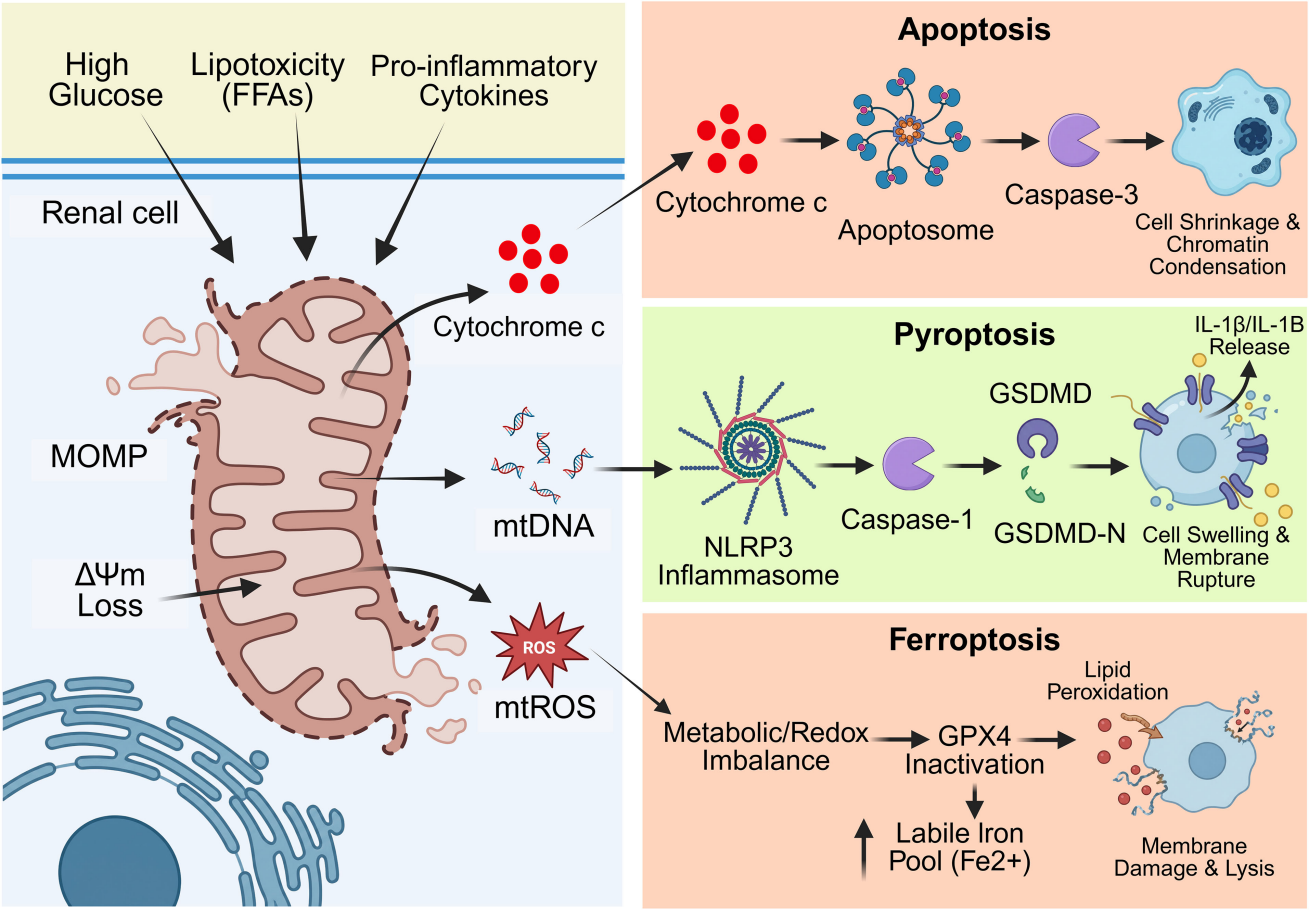
Mitochondrial dysfunction as the central signaling hub for RCD in diabetic kidney disease. (Left) Upstream metabolic and inflammatory stressors (high glucose, lipotoxicity, cytokines) converge to compromise mitochondrial integrity, leading to membrane permeabilization and the release of critical danger signals: cytochrome c, mitochondrial DNA (mtDNA), and reactive oxygen species (mtROS). (Right) These mitochondrial effectors distinctively activate downstream RCD cascades: cytosolic cytochrome c initiates apoptosis, leaked mtDNA triggers NLRP3 inflammasome-mediated pyroptosis, and ROS accumulation fuels ferroptosis via lipid peroxidation. RCD, regulated cell death; FFAs, free fatty acids; MOMP, mitochondrial outer membrane permeabilization; NLRP3, NOD-like receptor family pyrin domain containing 3; GSDMD, gasdermin D; GPX4, glutathione peroxidase 4.

The expression of the main mitophagy regulators, such as PTEN-induced kinase 1 (a mitochondrial serine/threonine kinase) and Parkin (an E3 ubiquitin ligase), is significantly suppressed in high-glucose environments and in models of DKD (Wu et al., 2025). The PTEN-induced kinase 1/Parkin axis is needed to specifically clear defective mitochondria (Fan et al., 2024). The impairment of this pathway causes dysfunctional mitochondria to evade clearance and consequently accumulate within renal cells. Such mitochondrial dysfunction can diverge into various RCD programs, which can help explain the breadth and richness of DKD pathology. Theoretically, optimal therapeutic interventions should target upstream mitochondrial dysfunction to simultaneously regulate multiple downstream RCD pathways (Li et al., 2025). As illustrated in **Figure 1**, the mitochondria-driven RCD network in DKD integrates apoptosis, pyroptosis, and ferroptosis through shared mitochondrial signaling nodes.

### 2.1 Apoptosis

Intrinsic apoptosis is an apparent RCD subtype in the DKD pathology. Prolonged hyperglycemia leads to the disruption in the balance between pro and anti-apoptotic B-cell lymphoma 2 (Bcl- 2) family proteins and results in the up-regulation of Bcl-2-associated X protein (Bax) and translocation to the outer membrane of mitochondria (Zhang et al., 2019). This oligomerization of Bax results in the execution of the mitochondrial outer membrane permeabilization event which is a decisive event, as it commits the cell to death by allowing the release of cytochrome c into the cytosol. Cytochrome c in turn forms an apoptosome with apoptotic protease activating factor-1 and pro-caspase-9 and initiates a caspase-9/caspase-3 cascade that results in DNA fragmentation and chromatin condensation (Yang et al., 2023). It is important to mention that besides the metabolic stimuli that cause the activation of mitochondrial apoptosis, the difference between the renal cell types is the most fundamental. The early DKD is characterized by metabolic reprogramming of proximal tubular epithelial cells, which normally depend almost exclusively on fatty acid oxidation to supply high energy levels, and their metabolic switch to glycolysis. Although this glycolytic shift may be autonomous of fatty acid oxidation defects and be an initial adaptive response to relative hypoxia, the ultimate result of the gradual provision of fatty acid oxidation together with the elevated uptake of lipids through CD36 and FATP2 is intra-cellular lipid accumulation (lipotoxicity) and ATP depletion. The net result of this metabolic imbalance is the direct overstimulation of the electron transport chain resulting in the production of massive mitochondrial ROS and the development of oxidative stress. In contrast, podocytes express GLUT4 and are sensitive to insulin, relying on glucose uptake for metabolism. Under hyperglycemic conditions, glucose overload and dysregulated insulin signaling induce massive ROS production via mitochondrial dysfunction and activate the Nrf2 pathway (Mohandes et al., 2023). Moreover, the cytotoxicity of the podocytes specifically appears in the form of cholesterol-enriched lipid droplet-based accumulation and ER stress that intermingle with the mitochondria to enhance the death signal (Shen et al., 2022). In line with these separate though convergent processes, diabetic renal tubules display a heightened proportion of Bax to Bcl-2 and caspase-3 activation, confirming that the mitochondrial apoptosis is an essential executioner of the podocyte and tubular epithelial cell obliteration.

### 2.2 Pyroptosis

Distinct from apoptosis, pyroptosis manifests as a highly inflammatory lytic cascade driven by the activation of the NOD-like receptor family pyrin domain containing 3 (NLRP3) inflammasome. Mitochondrial dysfunction induced under chronic glucose-overload and oxidative-stress conditions produces excess ROS and releases mitochondrial DNA (mtDNA) to the cytosol which are potent triggers of NLRP3 assembly. Upon activation, the NLRP3 inflammasome cleaves pro–caspase-1 into active caspase-1, which in turn processes pro–IL-1β and pro–IL-18 into their mature cytokines and cleaves gasdermin D (Zheng et al., 2023). This causes the N-terminal fragment of gasdermin D to create plasma membrane pores, and this causes cell swelling, lysis, and the release of inflammatory mediators. As a result, pyroptosis enhances local inflammation and fibrotic remodeling in DKD (Chen et al., 2025). However, it is worth noting that while experimental models firmly establish this cascade, evidence in human DKD biopsies remains largely indirect. Current human transcriptomic data implicate pyroptosis-related markers, but robust single-cell confirmation of gasdermin or caspase-1 executioner signatures in human kidneys is still limited, highlighting the need for deeper clinical validation (Acoba et al., 2025).

### 2.3 Ferroptosis

Mitochondrial redox imbalance and unregulated lipid peroxidation were the major causes of ferroptosis (Jia et al., 2024). Metabolic reprogramming in DKD switches cellular energy generation between fatty acid oxidation to glycolysis that leads to build-up of polyunsaturated fatty acids (Mu et al., 2025). Reduced fatty acid oxidation is another factor that leads to escalation of lipid metabolism and redox imbalance in the mitochondrion that triggers inflammatory cascades and kidney tubular damage (Sun et al., 2025). Simultaneously, hyperglycemia attenuates the glutathione/glutathione peroxidase 4 (GPX4) lipid-repairing enzyme undermining cell survival in lipid peroxidation processes (Kanikarla-Marie et al., 2019). Crucially, these experimental findings are directly corroborated by human biopsy transcriptomics, which reveal significantly reduced mRNA levels of canonical anti-ferroptosis genes in the renal tubules of DKD patients compared to non-diabetic controls (Liu et al., 2024a). The combination of GPX4 depletion, polyunsaturated fatty acid saturation, and labile iron accumulation leads to uninhibited lipid peroxidation and membrane injury. Morphologically, this is characterized by shrunken mitochondria with condensed membranes, underscoring the core role of this organelle in ferroptosis (Liu et al., 2024b).

### 2.4 Crosstalk among RCD pathways: a mitochondrial death network

Current evidence indicates that apoptosis, pyroptosis, and ferroptosis in DKD do not follow a strict chronological sequence (such as exclusively early apoptosis versus late ferroptosis). Instead, they are co-activated, highly overlapping, and exhibit distinct cell-type preferences. Podocyte loss is predominantly driven by apoptosis (Yang et al., 2024), whereas proximal tubular epithelial cells demonstrate a pronounced susceptibility to ferroptosis and pyroptosis, which strongly drive subsequent tubulointerstitial fibrosis (Bai et al., 2024; Zhou et al., 2023).

These pathways are linked through both direct molecular crosstalk and functional interdependence. For instance, Caspase-3, historically an apoptotic executioner, can act as a molecular switch in renal tubules. Under stress, activated Caspase-3 cleaves gasdermin E, liberating its necrotic N-terminal fragment to form membrane pores, thereby converting non-inflammatory apoptosis into secondary pyroptosis. Furthermore, while a direct molecular switch between canonical ferroptosis and pyroptosis proteins remains unidentified, they are profoundly intertwined via shared inflammatory circuits. The lipid peroxidation, iron overload, and mitochondrial DAMPs (such as mtDNA) released during ferroptosis secondarily activate the NLRP3 inflammasome, creating a mutually reinforcing loop between oxidative injury and pyroptotic inflammation (Wang et al., 2017).

The tumor suppressor p53 further orchestrates this network, functioning as a binary switch that promotes apoptosis via Bax while simultaneously facilitating ferroptosis through the repression of solute carrier family 7 member 11 (SLC7A11) (Liu and Gu, 2022). Together, these findings depict mitochondrial dysfunction not as a singular executioner, but as the apex trigger for a spectrum of parallel, interdependent cell death modes. Importantly, while bulk transcriptomics of human DKD biopsies robustly confirm severe mitochondrial dysfunction (e.g., downregulated oxidative phosphorylation) and support tubular ferroptosis signatures, current single-cell and single-nucleus RNA sequencing (sc/snRNA-seq) atlases have not yet unequivocally resolved these overlapping execution programs at the single-cell level (Tan et al., 2024). This indicates that while the network perspective is conceptually strong, mapping its exact spatiotemporal dynamics in humans remains a critical translational frontier. Given the complexity of these interactions, we have summarized the key crosstalk nodes and their specific phytochemical modulators in **Table 1**.

**Table 1:** Key crosstalk nodes and phytochemical modulators in the mitochondrial RCD network.

Crosstalk node	Pathway interaction	Core mechanism	Phytochemical modulators
Caspase-3/GSDME	Apoptosis → Pyroptosis	Cleaves GSDME to form pores, shifting death to an inflammatory mode.	Berberine, Curcumin, Puerarin
mtROS/mtDNA	Dysfunction → Pyroptosis	Leaked ROS and DNA act as DAMPs to trigger NLRP3 inflammasome assembly.	Luteolin, Curcumin
p53	Apoptosis → Ferroptosis	Dual switch: induces apoptosis (via Bax) and facilitates ferroptosis (represses SLC7A11).	Resveratrol
GPX4/Lipid ROS	Ferroptosis →Pyroptosis	Lipid peroxidation releases DAMPs that secondarily activate NLRP3.	Quercetin, Berberine
Bcl-2/Bax	Mitochondrial Integrity → Apoptosis	Controls MOMP and Cytochrome c release.	Berberine, Curcumin

GSDME, gasdermin E; mtROS, mitochondrial reactive oxygen species; mtDNA, mitochondrial DNA; NLRP3, NOD-like receptor family pyrin domain containing 3; DAMPs, damage-associated molecular patterns; SLC7A11, solute carrier family 7 member 11; MOMP, mitochondrial outer membrane permeabilization..

## 3 Phytochemicals

Key compounds, molecular targets, mechanisms of action, and primary renal protective effects are summarized in **Table 2**.

**Table 2:** Representative phytochemicals targeting RCD pathways in preclinical models of DKD.

Targeted RCD pathway	Phytochemical	Experimental model (cell or animal)	Administration and dosage	Key molecular targets	Core mechanism	Renal protective effects	Reference
Inhibition of Apoptosis	Berberine	*In vitro*: 1. Rat proximal tubular cells (NRK-52E) and Human HK-2 cells (HG 30 mM) 2. Mouse podocytes (MPC-5) (AGEs 50 µg/mL)	*In vitro*: 1. 20–30 µM (co-treatment/pretreatment) for 24 h 2. 5 µM pretreatment for 6 h	Sirt1, FoxO3a, Bnip3, PI3K, Akt, Nrf2, HO-1, PKM2, Bcl-2, Bax.	Activates Sirt1/FoxO3a/Bnip3 axis to induce mitophagy; activates PI3K/Akt/Nrf2 pathway to enhance antioxidant defense; upregulates PKM2 to restore mitochondrial dynamics.	Inhibits apoptosis in tubular cells and podocytes; restores mitochondrial membrane potential; reduces ROS generation and albuminuria.	(Zhang et al., 2016; Gong et al., 2024; Saxena et al., 2024)
	Resveratrol	*In vivo*: 1. db/db mice (Type 2 DN) 2. STZ-induced CD-1 mice (Type 1 DN) *In vitro*: Mouse GMCs, Podocytes, and HK-2 cells (HG 30–40 mM)	*In vivo*: 10–40 mg/kg/day (i.g.) for 12–16 weeks *In vitro*: 2.5–10 µM for 24–48 h; 5 µM for 5 days	SIRT1, PGC-1α, PDE4D, PKA, Drp1, p21, NRF1, TFAM	Inhibits PDE4D to activate PKA, phosphorylating Drp1 (Ser637) to block mitochondrial fission; activates SIRT1/PGC-1α axis to enhance mitochondrial biogenesis; suppresses senescence via SIRT1/p21 pathway.	Reduces apoptosis and senescence (SA-β-gal); inhibits mitochondrial fragmentation and ROS; alleviates renal fibrosis (collagen IV, laminin) and proteinuria.	(Zhang et al., 2019; Zhu et al., 2023; Xu et al., 2024)
	Curcumin	*In vivo*: STZ-induced Type 1 diabetic rats (Sprague-Dawley)	*In vivo*: 100 mg/kg/day (i.g.) for 12 weeks	PKCβ, p66Shc, FOXO-3a, Nrf2, Bax/Bcl-2, Caspase-3	Inhibits PKCβ/p66Shc pathway to reduce mtROS; activates FOXO3a/Nrf2; regulates Bcl-2 family.	Reduces oxidative stress and apoptosis, protects mitochondria, and improves renal function.	(Altamimi et al., 2021)
	Puerarin	*In vivo*: STZ-induced diabetic mice (C57BL/6J & Kunming) *In vitro*: Mouse podocytes (MPC-5) (HG 30 mM)	*In vivo*: 20–80 mg/kg/day (i.g.) for 8–12 weeks *In vitro*: 10–40 µM for 24 h	SIRT1, FOXO1, PGC-1α, HO-1, LKB1, AMPK	Activates SIRT1/FOXO1/PGC-1α axis to reduce oxidative stress; restores autophagy via HO-1/LKB1/AMPK signaling to inhibit apoptosis.	Reduces albuminuria (UACR) and BUN/Scr; attenuates glomerular mesangial expansion and preserves mitochondrial ultrastructure.	(Xu et al., 2016; Li et al., 2020)
Inhibition of Pyroptosis	Luteolin	*In vitro*: Mouse podocytes (MPC-5) exposed to High Glucose (30 mM)	*In vitro*: Pretreatment for 24 h (concentration-dependent)	mtROS, NLRP3, Caspase-1, IL-1β	Scavenges mtROS; directly inhibits NLRP3 inflammasome assembly and caspase-1 activation.	Reduces IL-1β/IL-18 release, alleviates renal inflammation, and protects renal cells from pyroptosis.	(Yu et al., 2019)
	Loganin	*In vivo*: HFD + STZ-induced diabetic mice (C57BL/6J) *In vitro*: Human HK-2 cells (HG 30 mM)	*In vivo*: 50, 100 mg/kg/day (i.p.) for 8 weeks *In vitro*: 20 µM for 24–48 h	NLRP3, Caspase-1, GSDMD, NF-κB, ROS, IL-1β, IL-18	Inhibits ROS generation to block NLRP3 inflammasome activation and the downstream Caspase-1/GSDMD pyroptotic pathway; also suppresses NF-κB.	Reduces serum/renal inflammatory cytokines (IL-1β, IL-18); ameliorates tubular injury and renal fibrosis; improves renal function (UACR, BUN, Scr).	(Kong et al., 2023)
Antagonism of Ferroptosis	Quercetin	*In vivo*: 1. STZ-induced diabetic rats (SD) 2. db/db mice (C57BL/KsJ) *In vitro*: Human HK-2 cells (HG 30 mM)	*In vivo*: 25–100 mg/kg/day (i.g.) for 12 weeks *In vitro*: 25–50 µM for 24–48 h	Nrf2, Keap1, GPX4, SLC7A11, FTH1, ACSL4, TFR-1	Activates Nrf2 pathway to upregulate GPX4/SLC7A11 (anti-lipid peroxidation) and FTH1 (iron storage); downregulates ACSL4 and TFR-1 to suppress ferroptosis.	Inhibits ferroptosis in renal tubular cells, reduces lipid peroxidation, and improves kidney function.	(Feng et al., 2023; Zhang et al., 2024)
	Berberine	*In vivo*: STZ-induced diabetic mice (C57BL/6) *In vitro*: Human HK-2 cells	*In vivo*: 100–200 mg/kg/day (i.g.) for 8 weeks *In vitro*: 50 µM	KLF4 (promoter methylation), DNMT1, GPX4, SLC7A11	Inhibits DNMT1 to prevent KLF4 promoter methylation; restored KLF4 transcriptionally activates SLC7A11 and GPX4 to suppress ferroptosis.	Inhibits ferroptosis (reduces Iron, ROS, MDA); alleviates renal fibrosis (Collagen, TGF-β1) and improves renal function.	(Cai et al., 2024)

DKD, diabetic kidney disease; HG, high glucose; AGEs, advanced glycation end products; SIRT1, silent information regulator 1; FOXO, forkhead box O; PI3K, phosphatidylinositol 3-kinase; Nrf2, nuclear factor erythroid 2–related factor 2; HO-1, heme oxygenase-1; PKM2, pyruvate kinase M2; PGC-1α, peroxisome proliferator-activated receptor-gamma coactivator-1alpha; PDE4D, phosphodiesterase 4D; Drp1, dynamin-related protein 1; TFAM, mitochondrial transcription factor A; PKCβ, protein kinase C beta; AMPK, AMP-activated protein kinase; IL, interleukin; NF-κB, nuclear factor kappa B; DNMT1, DNA methyltransferase 1; KLF4, Krüppel-like factor 4; TGF-β1, transforming growth factor-beta 1; MDA, malondialdehyde; BUN, blood urea nitrogen; Scr, serum creatinine; UACR, urinary albumin-to-creatinine ratio.

### 3.1 Inhibition of apoptosis by phytochemicals

Inhibition of apoptosis is one of the most classic and widely studied protective mechanisms of phytochemicals in DKD. These compounds usually act on the mitochondrial pathway controlled by Bcl-2 family proteins or activate endogenous pro-survival signals.

#### 3.1.1 Berberine

Berberine is an isoquinoline alkaloid extracted from Coptis species and other plants and functions as a multi-target inhibitor of apoptosis. Extensive research demonstrates that berberine intervention directly disrupts the mitochondrial apoptotic cascade (Ai et al., 2021). In DKD models, berberine restores impaired mitochondrial membrane potential and suppresses the pro-apoptotic protein Bax while upregulating the anti-apoptotic protein Bcl-2. This stabilizes the outer mitochondrial membrane, preventing cytochrome c release and the downstream caspase-9/caspase-3 cascade (Lv et al., 2012). Further investigations indicate that berberine also targets the non-coding transcriptome, specifically downregulating lncRNA *LOC102549726* to inhibit podocyte apoptosis (Wang et al., 2025). Beyond mitochondrial suppression, berberine promotes survival signaling via the PI3K/Akt axis, leading to the phosphorylation and inhibition of pro-apoptotic factors (Kim et al., 2014).

#### 3.1.2 Resveratrol

Resveratrol is a polyphenol found in grapes, Polygonum cuspidatum, and other plants, whose effective anti-apoptotic effects are mainly linked to activation of silent information regulator 1 (SIRT1), an NAD+-dependent deacetylase (Yun et al., 2012; Yacoub et al., 2014). In DKD models, resveratrol-induced SIRT1 deacetylates and inhibits the pro-apoptotic transcription factor p53, leading to decreased expression of p53 target genes (such as *Bax* and *PUMA*) and reduced apoptosis (Kim et al., 2011). Concurrently, SIRT1 activation deacetylates and activates peroxisome proliferator-activated receptor-gamma coactivator-1alpha (PGC-1α), a transcriptional co-activator and master regulator of mitochondrial biogenesis (Kitada et al., 2011). By enhancing mitochondrial biogenesis, resveratrol maintains mitochondrial health and indirectly suppresses apoptosis. This replenishment of healthy mitochondria represents a direct strengthening of MQC to counteract mitochondrial loss in DKD (Carollo et al., 2025).

#### 3.1.3 Curcumin

Curcumin is an anti-inflammatory polyphenol anti-oxidant of turmeric. It mechanistically operates by disrupting the Protein kinase C beta (PKCβ) dependent oxidative pathway. The activation of PKCβ under hyperglycemic conditions and the phosphorylation of the adaptor protein p66Shc result in the translocation of this protein to the mitochondria to trigger ROS. Curcumin attenuates mitochondrial oxidative stress by inhibiting this PKCβ/p66Shc signaling cascade (Sun et al., 2016). As a result, the stabilization of the mitochondrial membrane prevents the increase in the Bax/Bcl-2 ratio and caspase-3 activation induced by a high glucose level and preserves podocytes and tubular epithelial cells against apoptosis. Also, it enhances cell survival by stimulating the Forkhead box O3 transcription factor, that plays a role in oxidative and metabolic resistance (Altamimi et al., 2021).

#### 3.1.4 Puerarin

This isoflavone known to extract out of the rhizome of Pueraria has strong renoprotective action via the activation of the SIRT1-mediated signaling networks. It upregulates the SIRT1/FOXO1/PGC-1α axis to attenuate mitochondrial oxidative stress and inflammation in the diabetic kidney (Li et al., 2020). Furthermore, recent evidence indicates that puerarin restores podocyte autophagy via the HO-1/SIRT1/LKB1/AMPK pathway, thereby effectively inhibiting high glucose-induced apoptosis and preserving glomerular integrity (Xu et al., 2016).

### 3.2 Inhibition of pyroptosis by phytochemicals

Considering that pyroptosis is mostly inhibited through inhibiting NLRP3 inflammasome activation, this can be done by removing upstream activation factors (like mtROS) or by direct inhibition of inflammasome formation.

#### 3.2.1 Luteolin

Luteolin directly inhibits pyroptosis (Mahwish et al., 2025). It is a good mitochondrial ROS scavenger and its presence helps to inhibit the early arrangement of NLRP3 inflammasome. Luteolin inhibits caspase-1 activation and maturation of IL-1β oligomerizing and recruiting apoptosis-related speck-like protein with a CARD, an adaptor protein to underpin pyroptotic inflammation in DKD (Yu et al., 2019).

#### 3.2.2 Loganin

Loganin is an iridoid glycoside, which reduces kidney damage in DKD through the interaction with the NLRP3 inflammasome-mediated pyroptosis. Loganin suppresses the production of ROS in streptozotocin-induced diabetic mice and high glucose-treated HK-2 cells and, thus, blocks the NLRP3/Caspase-1/GSDMD pathway. This blockade suppresses the development and release of proinflammatory cytokines IL-1β and IL-18, which eventually suppresses the inflammation and fibrosis of renal tubular epithelial cells (Kong et al., 2023).

### 3.3 Antagonism of ferroptosis by phytochemicals

The new type of RCD, ferroptosis, is strongly suppressed by several phytochemicals, thus paving new opportunities in the therapeutic direction of DKD (Wu and Huang, 2024). These substances tend to accumulate in optimizing the innermost anti-lipid-peroxidation safeguarding framework: The nuclear factor erythroid 2-related factor 2 (Nrf2)-glutathione-GPX4.

#### 3.3.1 Quercetin

Quercetin is a flavonoid that is found in fruits and vegetables and a well-characterized ferroptotic inhibitor (Efiong et al., 2025). The major mechanism by which it works is through the activation of the Nrf2 pathway (Zhang et al., 2024). Nrf2 is a master regulator of antioxidant defenses: it is bound by Kelch-like ECH-associated protein 1 (Keap1), a repressor protein, targeted for degradation, but under oxidative or electrophilic pressure it dissociates from Keap1, translocates to the nucleus, and induces a battery of antioxidant and detoxification genes. Quercetin promotes Nrf2’s nuclear translocation and upregulates the expression of downstream targets, most critically the genes encoding *GPX4* and *SLC7A11*. By simultaneously increasing the core ferroptosis-defense enzyme and the capacity for glutathione synthesis, quercetin robustly enhances the cell’s resistance to lipid peroxidation (Feng et al., 2023). However, the chronic activation of Nrf2 requires critical assessment. Excessive Nrf2 signaling can induce “reductive stress” and paradoxically worsen podocyte injury, proteinuria, and renal fibrosis (Bondi et al., 2024). The translational risks are starkly highlighted by the clinical failure of potent Nrf2 activators like bardoxolone methyl in DKD due to severe cardiovascular toxicities (Lin et al., 2023). Furthermore, sustained systemic Nrf2 activation raises extra-renal concerns, including potential tumor promotion (Ngo et al., 2025). Therefore, the therapeutic viability of phytochemicals like quercetin relies entirely on their ability to provide transient, tissue-targeted, and tightly dose-controlled Nrf2 modulation.

#### 3.3.2 Berberine

Berberine’s multitarget nature is again evident in ferroptosis. Recent studies show that berberine not only inhibits apoptosis but also prevents ferroptosis by activating Nrf2 (Cai et al., 2024). In DKD models, berberine upregulates Nrf2 and its downstream genes (such as *GPX4* and heme oxygenase-1, *HO-1*), which leads to reduced renal iron deposition and lower levels of lipid peroxidation markers (Yang et al., 2022). This adds another important mechanistic dimension to berberine’s nephroprotective effects, making it a prototypical compound that can simultaneously intervene in multiple RCD pathways.

## 4 Discussion

While preclinical data provide a compelling framework, a critical evaluation of these models is necessary to address the translational gap. Most *in vivo* studies rely on streptozotocin (STZ)-induced models, which primarily mimic Type 1 diabetes through acute β-cell destruction. STZ models exhibit direct chemical nephrotoxicity and fail to capture the multifactorial complexity (such as obesity, insulin resistance) and advanced fibrotic lesions of human Type 2 DKD (Pointeau et al., 2025). Furthermore, rodent models provide only short-term snapshots of mitochondrial dysfunction, failing to reflect the decades-long systemic milieu characteristic of human disease (Hu et al., 2025). Similarly, *in vitro* single-cell models inherently lack the essential multicellular crosstalk, such as endothelial-podocyte communication, crucial for DKD progression (Casalena et al., 2020). Finally, a major confounding factor *in vivo* is the systemic metabolic impact of phytochemicals (Hu et al., 2022). Because agents like berberine significantly reduce systemic glucose and lipids, their suppression of RCD could be partially secondary to glycemic control. Although *in vitro* evidence confirms direct, cell-autonomous mitochondrial modulation, uncoupling these local effects from systemic benefits *in vivo* remains challenging. To conclusively isolate direct renal targeting, future preclinical studies must incorporate “glucose-matched” controls.

We present evidence that renal cell death in DKD operates not as a singular event, but as a complex network composed of apoptosis, pyroptosis, and ferroptosis, with mitochondrial dysfunction acting as the convergent hub that integrates upstream biochemical and oxidative insults to propagate the death cascade. The integrated RCD network model, which incorporates critical crosstalk points like the caspase-3/gasdermin E switch and the ferroptosis-damage signal-NLRP3 axis, provides a far more sophisticated framework for understanding both the heterogeneity of DKD and the pleiotropic effects of phytochemicals. The clinical relevance of this mitochondrial-RCD framework is strongly supported by the current standard of care, SGLT2 inhibitors. Preclinical models demonstrate that SGLT2 inhibitors directly modulate mitochondrial quality control and mitophagy while robustly inhibiting tubular ferroptosis by restoring GPX4 and SLC7A11 expression (Tian et al., 2025). Although their direct inhibition of pyroptosis remains mostly inferential, human metabolomic data corroborate that SGLT2 inhibitors successfully improve mitochondrial metabolism in patients (Guo et al., 2025). Therefore, benchmarking phytochemicals alongside SGLT2 inhibitors represents a crucial translational step to determine their complementary or additive renoprotective value.

Although preclinical studies show phytochemicals potently regulate mitochondrial pathways, severe pharmacokinetic limitations hinder their clinical translation. A critical dosing disconnect exists: *in vitro* mechanistic studies typically rely on micromolar concentrations, yet standard oral supplementation yields only low nanomolar plasma levels, an efficacy gap of up to three orders of magnitude. Compounds like curcumin, resveratrol, berberine, and quercetin are severely limited by poor solubility, extensive first-pass metabolism, and efflux transporters (Kong et al., 2025; Kroon et al., 2025; Radeva and Yoncheva, 2025). Consequently, achieving sufficient intracellular concentrations to engage mitochondrial nodes via traditional oral preparations is highly improbable. This barrier directly explains the contradictory clinical findings, where recent human trials often fail to significantly improve hard renal endpoints like eGFR or serum creatinine. Thus, translating these renoprotective effects urgently requires advanced delivery strategies (Gimblet et al., 2024).

Nanotechnology-based delivery systems have been proposed as one of the solutions to help bridge the gap between *in vitro* and clinical use. Innovative formulations, including liposomes, chitosan-functionalized solid lipid nanoparticles (SLNs) have been demonstrated to greatly enhance the stability and accumulation of phytochemicals including resveratrol and berberine in the kidney. Furthermore, the pathological microenvironment of DKD can be exploited for precision delivery: it is possible to tune nanocarrier size (30–80 nm) to passively accumulate in the mesangium, whereas altering the surface hydrophilicity by ligands (such as targeting megalin) or depending on ROS-activated linkers to ensure active internalization by proximal tubules and release the cargo precisely in the oxidative microenvironment. In addition to their therapeutic use, these targeted systems are also important critical mechanistic probes to selectively perturb mitochondrial nodes *in vivo*, and thus supporting the hypothesis of the mitochondria central hub proposed in this review.

## 5 Future perspectives

While the integration of liquid-liquid phase separation (LLPS) into the DKD paradigm remains a highly speculative hypothesis with no direct renal-specific experimental evidence to date, it offers a compelling biophysical framework for future research (Usman et al., 2025). Mitochondria intrinsically utilize LLPS to organize dynamic structures, such as mitochondrial nucleoids and RNA granules (Gao et al., 2025). Drawing on established LLPS paradigms in neurodegeneration, we postulate that the chronic oxidative stress and ATP depletion characteristic of DKD may drive aberrant “liquid-to-solid” phase transitions (Yoshizawa et al., 2020). This pathological biophysical hardening could sequester regulated cell death (RCD) initiators into irreversible aggregates and impede mitochondrial quality control mechanisms like mitophagy.

Crucially, this speculative framework provides a novel mechanistic lens for phytochemical action. Current biophysical evidence demonstrates that polyphenols, such as resveratrol and quercetin, can modulate biomolecular condensates and destabilize aberrant protein aggregations (such as amyloid-like assemblies) (Freyssin et al., 2018; Martins et al., 2023). Therefore, we hypothesize that these natural compounds might interact with intrinsically disordered regions of mitochondrial proteins to conserve condensate viscoelasticity, preventing the pathological solidification of stress-induced signaling hubs. Validating this aberrant mitochondrial phase behavior in human renal tissue represents an exciting, albeit nascent, frontier for DKD drug discovery.

## 6 Conclusion

Rather than interpreting apoptosis, pyroptosis, and ferroptosis as completely distinct entities, this work proposes a framework wherein these pathways operate as a highly interconnected network characterized by functional interdependence and spatiotemporal dynamics, governed by mitochondrial signaling. This perspective suggests that mitochondrial dysfunction acts as a critical upstream integrator in the progression of renal cell injury. Within this framework, phytochemicals demonstrate potential as network-level modulators to restore mitochondrial integrity, though their clinical translation requires overcoming bioavailability hurdles, potentially through nanotechnology-based delivery strategies. Furthermore, the incorporation of biophysical concepts, such as phase separation, offers an emerging lens to understand the spatial organization of death signals. Future research should focus on validating the pathological relevance of phase separation in human tissue and mapping cell-type specific mitochondrial networks. Merging biochemical signaling insights with biophysical organization principles may ultimately yield a more holistic paradigm for DKD drug discovery.

## Glossary

AMPK: AMP-activated protein kinase
Akt: Protein kinase B
Apaf-1: Apoptotic protease activating factor-1
ASC: Apoptosis-associated speck-like protein containing a CARD
ATP: Adenosine triphosphate
Bax: Bcl-2–associated X protein
Bcl-2: B-cell lymphoma 2
BUN: Blood urea nitrogen
CARD: Caspase recruitment domain
CD36: Cluster of differentiation 36
DKD: Diabetic kidney disease
DAMPs: Damage-associated molecular patterns
DFNA5 (GSDME): Gasdermin E
DMSO: Dimethyl sulfoxide
DNMT1: DNA methyltransferase 1
Drp1: Dynamin-related protein 1
eGFR: Estimated glomerular filtration rate
ER: Endoplasmic reticulum
FAO: Fatty acid oxidation
FATP2: Fatty acid transport protein 2
FOXO1: Forkhead box O1
FOXO3a: Forkhead box O3a
FTH1: Ferritin heavy chain 1
GLUT4: Glucose transporter type 4
GMCs: Glomerular mesangial cells
GPX4: Glutathione peroxidase 4
GSDMD: Gasdermin D
HG: High glucose
HK-2: Human kidney-2 proximal tubular epithelial cells
HO-1: Heme oxygenase 1
IL-1β: Interleukin-1 beta
IL-18: Interleukin-18
Keap1: Kelch-like ECH-associated protein 1
KLF4: Krüppel-like factor 4
LKB1: Liver kinase B1
LLPS: Liquid–liquid phase separation
MDA: Malondialdehyde
MOMP: Mitochondrial outer membrane permeabilization
MQC: Mitochondrial quality control
mtDNA: Mitochondrial DNA
NF-κB: Nuclear factor kappa B
NLRP3: NOD-like receptor family pyrin domain-containing 3
Nrf2: Nuclear factor erythroid 2-related factor 2
PDE4D: Phosphodiesterase 4D
PGC-1α: Peroxisome proliferator-activated receptor gamma coactivator 1-alpha
PI3K: Phosphatidylinositol 3-kinase
PKC: Protein kinase C
PKM2: Pyruvate kinase M2
PTEN: Phosphatase and tensin homolog
PUFAs: Polyunsaturated fatty acids
RAAS: Renin–angiotensin–aldosterone system
RCD: Regulated cell death
ROS: Reactive oxygen species
sc/snRNA-seq: Single-cell and single-nucleus RNA sequencing
Scr: Serum creatinine
SGLT2: Sodium-glucose cotransporter 2
SIRT1: Silent information regulator 1
SLC7A11: Solute carrier family 7 member 11
SLNs: Solid lipid nanoparticles
STZ: Streptozotocin
TCA cycle: Tricarboxylic acid cycle
TFAM: Mitochondrial transcription factor A
TGF-β1: Transforming growth factor-beta 1
UACR: Urinary albumin-to-creatinine ratio.
